# Risk modifiers of acute respiratory distress syndrome in patients with non-pulmonary sepsis: a retrospective analysis of the FORECAST study

**DOI:** 10.1186/s40560-020-0426-9

**Published:** 2020-01-10

**Authors:** Hiroki Iriyama, Toshikazu Abe, Shigeki Kushimoto, Seitaro Fujishima, Hiroshi Ogura, Atsushi Shiraishi, Daizoh Saitoh, Toshihiko Mayumi, Toshio Naito, Akira Komori, Toru Hifumi, Yasukazu Shiino, Taka-aki Nakada, Takehiko Tarui, Yasuhiro Otomo, Kohji Okamoto, Yutaka Umemura, Joji Kotani, Yuichiro Sakamoto, Junichi Sasaki, Shin-ichiro Shiraishi, Kiyotsugu Takuma, Ryosuke Tsuruta, Akiyoshi Hagiwara, Kazuma Yamakawa, Tomohiko Masuno, Naoshi Takeyama, Norio Yamashita, Hiroto Ikeda, Masashi Ueyama, Satoshi Fujimi, Satoshi Gando, on behalf of JAAM FORECAST group, Osamu Tasaki, Yasumitsu Mizobata, Hiraku Funakoshi, Toshiro Okuyama, Iwao Yamashita, Toshio Kanai, Yasuo Yamada, Mayuki Aibiki, Keiji Sato, Susumu Yamashita, Susumu Yamashita, Kenichi Yoshida, Shunji Kasaoka, Akihide Kon, Hiroshi Rinka, Hiroshi Kato, Hiroshi Okudera, Eichi Narimatsu, Toshifumi Fujiwara, Manabu Sugita, Yasuo Shichinohe, Hajime Nakae, Ryouji Iiduka, Mitsunobu Nakamura, Yuji Murata, Yoshitake Sato, Hiroyasu Ishikura, Yasuhiro Myojo, Yasuyuki Tsujita, Kosaku Kinoshita, Hiroyuki Yamaguchi, Toshihiro Sakurai, Satoru Miyatake, Takao Saotome, Susumu Yasuda, Toshikazu Abe, Hiroshi Ogura, Yutaka Umemura, Atsushi Shiraishi, Shigeki Kushimoto, Daizoh Saitoh, Seitaro Fujishima, Junichi Sasaki, Toshihiko Mayumi, Yasukazu Shiino, Taka-aki Nakada, Takehiko Tarui, Toru Hifumi, Yasuhiro Otomo, Joji Kotani, Yuichiro Sakamoto, Shin-ichiro Shiraishi, Kiyotsugu Takuma, Ryosuke Tsuruta, Akiyoshi Hagiwara, Kazuma Yamakawa, Naoshi Takeyama, Norio Yamashita, Hiroto Ikeda, Yasuaki Mizushima, Satoshi Gando

**Affiliations:** 1 Department of General Medicine, Juntendo University, 2-1-1 Hongo, 103 Bunkyo-ku, Tokyo, 113-0033 Japan; 2 Health Services Research and Development Center, University of Tsukuba, Tsukuba, Japan; 3 Department of Health Services Research, Faculty of Medicine, University of Tsukuba, Tsukuba, Japan; 4 Division of Emergency and Critical Care Medicine, Tohoku University Graduate School of Medicine, Sendai, Japan; 5 Center for General Medicine Education, Keio University School of Medicine, Tokyo, Japan; 6 Department of Traumatology and Acute Critical Medicine, Osaka University Graduate School of Medicine, Suita, Japan; 7 Emergency and Trauma Center, Kameda Medical Center, Kamogawa, Japan; 8 Division of Traumatology, Research Institute, National Defense Medical College, Tokyo, Japan; 9 Department of Emergency Medicine, School of Medicine, University of Occupational and Environmental Health, Kitakyushu, Japan; 10 Department of Emergency and Critical Care Medicine, St. Luke’s International Hospital, Tokyo, Japan; 11 Department of Acute Medicine, Kawasaki Medical School, Kurashiki, Japan; 12 Department of Emergency and Critical Care Medicine, Chiba University Graduate School of Medicine, Chiba, Japan; 13 Department of Trauma and Critical Care Medicine, Kyorin University School of Medicine, Mitaka, Japan; 14 Trauma and Acute Critical Care Center, Medical Hospital, Tokyo Medical and Dental University, Tokyo, Japan; 15 Department of Surgery, Center for Gastroenterology and Liver Disease, Kitakyushu City Yahata Hospital, Kitakyushu, Japan; 16 Department of Disaster and Emergency Medicine, Kobe University Graduate School of Medicine, Kobe, Japan; 17 Emergency and Critical Care Medicine, Saga University Hospital, Saga, Japan; 18 Department of Emergency and Critical Care Medicine, Keio University School of Medicine, Tokyo, Japan; 19 Department of Emergency and Critical Care Medicine, Aizu Chuo Hospital, Aizuwakamatsu, Japan; 20 Emergency & Critical Care Center, Kawasaki Municipal Kawasaki Hospital, Kawasaki, Japan; 21 Advanced Medical Emergency & Critical Care Center, Yamaguchi University Hospital, Ube, Japan; 22 Department of Emergency Medicine, Niizashiki Chuo General Hospital, Niiza, Japan; 23 Division of Trauma and Surgical Critical Care, Osaka General Medical Center, Osaka, Japan; 24 Department of Emergency and Critical Care Medicine, Nippon Medical School, Tokyo, Japan; 25 Advanced Critical Care Center, Aichi Medical University Hospital, Nagakute, Japan; 26 Advanced Emergency Medical Service Center, Kurume University Hospital, Kurume, Japan; 27 Department of Emergency Medicine, Teikyo University School of Medicine, Tokyo, Japan; 28 Department of Trauma, Critical Care Medicine, and Burn Center, Japan Community Healthcare Organization, Chukyo Hospital, Nagoya, Japan; 29 Division of Acute and Critical Care Medicine, Hokkaido University Graduate School of Medicine, Sapporo, Japan; 30 Department of Acute and Critical Care Medicine, Sapporo Higashi Tokushukai Hospital, Sapporo, Japan

**Keywords:** Acute respiratory distress syndrome, acute respiratory failure, sepsis

## Abstract

**Background:**

Predisposing conditions and risk modifiers instead of causes and risk factors have recently been used as alternatives to identify patients at a risk of acute respiratory distress syndrome (ARDS). However, data regarding risk modifiers among patients with non-pulmonary sepsis is rare.

**Methods:**

We conducted a secondary analysis of the multicenter, prospective, Focused Outcomes Research in Emergency Care in Acute Respiratory Distress Syndrome, Sepsis and Trauma (FORECAST) cohort study that was conducted in 59 intensive care units (ICUs) in Japan during January 2016–March 2017. Adult patients with severe sepsis caused by non-pulmonary infection were included, and the primary outcome was having ARDS, defined as meeting the Berlin definition on the first or fourth day of screening. Multivariate logistic regression modeling was used to identify risk modifiers associated with ARDS, and odds ratios (ORs) and their 95% confidence intervals were reported. The following explanatory variables were then assessed: age, sex, admission source, body mass index, smoking status, congestive heart failure, chronic obstructive pulmonary disease, diabetes mellitus, steroid use, statin use, infection site, septic shock, and acute physiology and chronic health evaluation (APACHE) II score.

**Results:**

After applying inclusion and exclusion criteria, 594 patients with non-pulmonary sepsis were enrolled, among whom 85 (14.3%) had ARDS. Septic shock was diagnosed in 80% of patients with ARDS and 66% of those without ARDS (*p* = 0.01). APACHE II scores were higher in patients with ARDS [26 (22–33)] than in those without ARDS [21 (16–28), *p* < 0.01]. In the multivariate logistic regression model, the following were independently associated with ARDS: ICU admission source [OR, 1.89 (1.06–3.40) for emergency department compared with hospital wards], smoking status [OR, 0.18 (0.06–0.59) for current smoking compared with never smoked], infection site [OR, 2.39 (1.04–5.40) for soft tissue infection compared with abdominal infection], and APACHE II score [OR, 1.08 (1.05–1.12) for higher compared with lower score].

**Conclusions:**

Soft tissue infection, ICU admission from an emergency department, and a higher APACHE II score appear to be the risk modifiers of ARDS in patients with non-pulmonary sepsis.

## Background

Acute respiratory distress syndrome (ARDS) comprises heterogenous clinical conditions. Reportedly, the prognosis of ARDS is poor [1, 2], and once a patient develops ARDS, treatment options are limited to only a few supportive strategies [3–9], making it important to identify patients at a high risk of ARDS [10]. Previous studies have reported a variety of causes and risk factors of ARDS [1, 11]; however, there is a lack of clarity between these because only a small proportion of patients with these causes and risk factors develop ARDS [12, 13]. Therefore, alternative efforts have been recently focusing on the roles of two types of risk factors: predisposing conditions and risk modifiers [14, 15]. Predisposing conditions are preceding acute pathophysiological events, such as sepsis. Risk modifiers include obesity [15, 16], smoking status [15, 17, 18], diabetes mellitus (DM) (reduced risk modifier) [14, 15, 19], glucocorticoids [20], statin [21, 22], non-pulmonary infection (reduced risk modifier) [13, 23], shock [13, 15], tachypnea [14, 15], oxygen supplementation [15, 24], hematocrit [11], hypoalbuminemia [14, 15], acidemia [11, 15], and disease severity [2, 11, 13]. There is a possibility that ARDS may be precisely predicted using a combination of predisposing conditions and risk modifiers.

ARDS has been associated with two major pathophysiologic changes in various proportions. One is the influx of protein-rich effusion to the alveolar space caused by the damage of the local alveolar epithelium and another is leakage to the pulmonary interstitium through the capillary endothelium caused by systemic inflammation. Direct ARDS is associated with higher impairment of alveolar epithelium and lower impairment of capillary endothelium than indirect ARDS [23, 25]. Thus, we think risk modifiers of direct and indirect ARDS should be discussed separately.

Indeed to date, however, little has been reported about risk modifiers for ARDS among patients with non-pulmonary sepsis because a large proportion of patients with pulmonary sepsis have been included in previous studies about risk modifiers [2, 14, 15].

We aimed to evaluate the risk modifiers associated with indirect ARDS among patients with non-pulmonary sepsis.

## Methods

### Design and setting

We conducted a secondary analysis of the sepsis cohort in the Focused Outcomes Research in Emergency Care in Acute Respiratory Distress Syndrome, Sepsis, and Trauma (FORECAST) study. This was a multicenter prospective cohort study of 1184 patients with severe sepsis or septic shock enrolled from 59 Intensive care units (ICUs) in Japan and conducted from January 2016 to March 2017 [26].

### Participants

We included adult patients from the FORECAST database if they were aged ≥ 16 years and had severe sepsis or septic shock caused by non-pulmonary infection. The exclusion criteria were patients with missing data of the first or fourth days of ARDS screening in this study.

### Data collection

Patient information was obtained from the FORECAST database, including demographic data, admission source, comorbidities, infection sites, sepsis-related severity scores, and laboratory data. Data collection was performed as part of the routine clinical workup by the original FORECAST investigators.

### Data definitions

ARDS was diagnosed if present on the first or fourth day of ARDS screening, according to the Berlin ARDS definition [27]. Severe sepsis and septic shock were defined based on the sepsis-2 criteria [28]. Non-pulmonary infection was defined as infection other than pneumonia or empyema. Cases of DM with and without end-organ complications were reported as comorbidities. Also, “ventilator-free days” was defined as the number of days within the first 28 days after enrolment, during which a patient was able to breathe without the help of a ventilator. Patients who died during the study were assigned a ventilator-free day of 0. ICU-free days were calculated and scored in a similar manner [29].

### Statistical analysis

Patients were stratified into groups with and without ARDS (i.e., ARDS and No ARDS groups). Descriptive statistics were calculated as proportions for categorical variables and as medians (interquartile range [IQR]) or mean ± standard deviation (SD) for continuous variables, where appropriate. Statistical differences between two groups were evaluated by univariate analyses, using the chi-square or Fisher exact tests for categorical variables and the Mann–Whitney *U* test for continuous variables because the data did not have a normal distribution.

To identify the risk modifiers correlated with having ARDS in patients with non-pulmonary sepsis, we developed a multivariate logistic regression model and reported odds ratios (ORs) with their 95% confidence intervals (CIs). We hypothesized that there could be different risk modifiers for indirect ARDS compared with those for direct ARDS reported in previous studies. The primary outcome of interest was having ARDS, and the explanatory variables were selected based on previous research: body mass index, smoking status, DM, glucocorticoids, statin, site of infection, septic shock, and acute physiology and chronic health evaluation II (APACHE II) score. We also include clinically relevant explanatory variables, such as age, gender, admission source, and coexisting conditions (e.g., congestive heart failure and chronic obstructive pulmonary disease). However, we did not take variables such as tachypnea, oxygen supplementation, acidosis, and hypoalbuminemia into the logistic regression model because these possible risk modifiers might result from ARDS. Finally, the non-pulmonary Sequential Organ Failure Assessment (SOFA) score was used in a sensitivity analysis.

All *p* values were two-sided, with *p* values < 0.05 considered statistically significant. All statistical analyses were performed using the EZR software (Version 1.32) [30].

## Results

**Fig. 1:**
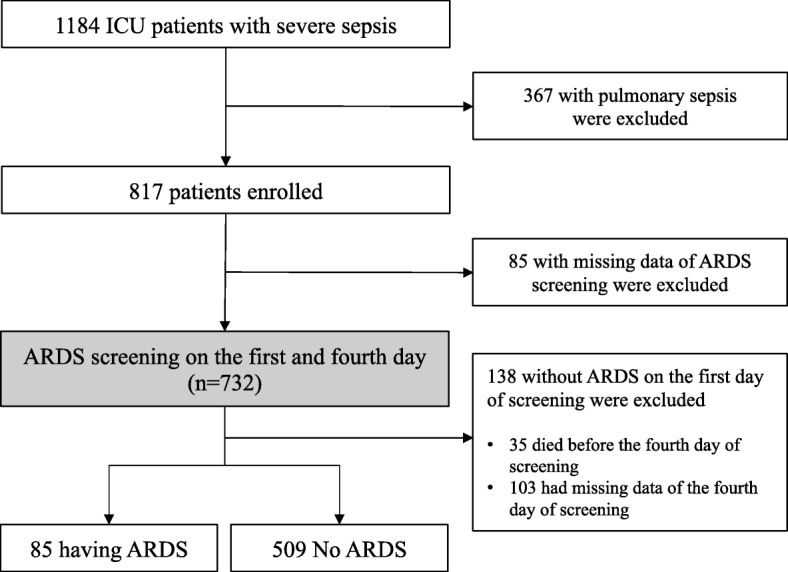
Flow of patient screening and enrolment ICU intensive care unit, ARDS acute respiratory distress syndrome.

Of the 1184 patients with severe sepsis in the FORECAST study, 817 with non-pulmonary infection were eligible for this study. Another 85 patients were excluded because they had missing data of the first day of ARDS screening. This left a cohort of 69 patients with ARDS and 663 without ARDS on the first day of screening. Of those without ARDS, 35 died on the second or third day and 103 patients had missing data of the fourth day of ARDS screening, so were excluded. Finally, 594 patients with non-pulmonary sepsis were enrolled, among whom 85 (14.3%) had ARDS at the first or fourth day of ARDS screening (the ARDS group) (Fig. 1)

### Baseline characteristics

The median age was 72 (IQR: 62–81) years and males accounted for 340 patients (57.2%). There were 231 patients (38.9%) with abdominal infection, 147 (24.7%) with urinary tract infection, and 91 (15.3%) with soft tissue infection. The baseline characteristics are compared between patients with and without ARDS in Table 1.

**Table 1:** Demographic, infection, and admission characteristics comparing those had ARDS and those did not

Characteristics		ARDS (*n* = 85)	No ARDS (*n* = 509)	*P* value
Age at admission—year		70 (62–80)	72 (63–82)	0.46
Male gender		51 (60)	289 (56.8)	0.66
Admission source	Hospital wards and other hospitals	28 (32.9)	226 (44.5)	0.12
	Emergency department	52 (61.2)	255 (50.2)	
	Intensive care unit	5 (5.9)	27 (5.3)	
Body mass index—kg/m^2^		23.4 ± 4.82	23.4 ± 5.45	0.07
Coexisting conditions	Myocardial infarction	5 (5.9)	27 (5.3)	0.80
	Congestive heart failure	9 (10.6)	53 (10.4)	1.00
	Peripheral vascular disease	2 (2.4)	10 (2)	0.69
	Cerebrovascular disease	7 (8.2)	65 (12.8)	0.31
	Dementia	4 (4.7)	44 (8.6)	0.28
	COPD	4 (4.7)	25 (4.9)	1.00
	Connective tissue disease	5 (5.9)	32 (6.3)	1.00
	Peptic ulcer disease	2 (2.4)	17 (3.3)	1.00
	Diabetes mellitus	21 (24.7)	121 (23.8)	0.96
	Chronic kidney disease	5 (5.9)	39 (7.7)	0.66
	Hemiplegia	2 (2.4)	20 (3.9)	0.76
	Malignancy (solid)	6 (7.1)	68 (13.4)	0.15
	Malignancy (blood)	1 (1.2)	9 (1.8)	1.00
	Metastatic tumor	2 (2.4)	15 (2.9)	1.00
	Mild liver disease	3 (3.5)	25 (4.9)	0.78
	Moderate to severe liver disease	0 (0)	15 (2.9)	0.15
	AIDS	0 (0)	1 (0.2)	1.00
CCI w/o diabetes mellitus		0 (0–1)	1 (0–2)	0.004
Smoking	Never	55 (66.3)	275 (59.0)	0.05
	Former	24 (28.9)	123 (26.4)	
	Current	4 (4.8)	68 (14.6)	
Regular medication	Glucocorticoids	9 (10.6)	69 (13.6)	0.56
	Immunosuppressants	3 (3.5)	21 (4.1)	1.00
	Anticoagulant	13 (15.3)	42 (8.3)	0.04
	Antiplatelet	14 (16.5)	87 (17.1)	1.00
	Statin	5 (5.9)	53 (10.4)	0.24
	Beta blocker	5 (5.9)	36 (7.1)	0.82
	Anticancer drug	2 (2.4)	15 (2.9)	1.00
	Antibiotics	16 (18.8)	70 (13.8)	0.29
Site of infection	Abdomen	32 (37.6)	199 (39.1)	0.25
	Urinary tract	15 (17.6)	132 (25.9)	
	Soft tissue	17 (20.0)	74 (14.5)	
	Other than abdomen, urinary tract, or soft tissue	21 (24.7)	104 (20.4)	
Septic shock		68 (80)	336 (66)	0.02
Respiratory rate—/min		25 (22–32)	24 (21–30)	0.34
FiO_2_		0.4 (0.3–0.5)	0.3 (0.25–0.4)	< 0.001
PaO_2_—mmHg		83 (75–103)	94 (78.6–112)	0.05
PaO_2_/FiO_2_ ratio		179 (119–240)	284 (184–374)	< 0.001
Hematocrit—%		33 (28–38)	33 (29–39)	0.38
Serum albumin—g/dL		2.4 (2.0–2.8)	2.6 (2.2–3.2)	0.01
Blood pH		7.36 (7.29–7.44)	7.41 (7.34–7.47)	0.003
Positive blood culture		54 (63.5)	303 (59.9)	0.53
Pathogens (blood culture)	Gram positive coccus	23 (27.1)	116 (22.8)	0.39
	Gram negative rod	28 (32.9)	167 (32.8)	0.98
APACHE II score		26 (21–33)	21 (16–28)	< 0.001
Non-pulmonary SOFA score		9 (7–11)	7 (4–9)	< 0.001

Reported counts (proportions) for categorical variables and median (interquartile range) for continuous variables

*ARDS* acute respiratory distress syndrome defined by Berlin criteria, *COPD* chronic obstructive pulmonary disease, *AIDS* acquired immune deficiency syndrome, *CCI* Charlson Comorbidity Index, *FiO*_2_ fraction of inspiratory oxygen, *PaO*_2_ partial pressure of oxygen in arterial blood, *APACHE II* acute physiology and chronic health evaluation II, *SOFA* sequential organ failure assessment

Definition of categorical variables: other than abdomen, urinary tract, or soft tissue = central nervous system, intravenous catheter, osteoarticular, endocardium, wound, implant device, and others; positive blood culture = culture without clinically confirmed contamination; Gram-positive coccus = *Staphylococcus*, *Streptococcus*, and *Enterococcus*; Gram-negative rod = *Acinetobacter*, *Aeromonas*, *Burkholderia*, *Citrobacter*, *Escherichia*, *Enterobacter*, *Haemophilus*, *Klebsiella*, *Legionella*, *Pseudomonas*, *Proteus*, *Salmonella*, *Serratia*, *Stenotrophomonas*, and *Vibrio*

Missing data (due to missing data of each outcome measures): admission source = 1; body mass index = 9; smoking = 45; respiratory rate = 1; FiO_2_ = 14; PaO_2_ = 18; PaO_2_/FiO_2_ ratio = 19; serum albumin = 10; blood pH = 14; positive blood culture = 3; APACHE II score = 53; non-pulmonary SOFA score = 46

Patients with ARDS had a lower Charlson Comorbidity Index than patients without ARDS, but there were no significant differences between the groups regarding other baseline characteristics, such as age, gender, and admission source. There was no significant difference between patients with and without ARDS regarding previously known risk modifiers for direct and indirect ARDS, including body mass index, DM, smoking status, and site of infection. A higher proportion of patients had septic shock with ARDS (80%) than without ARDS (66%; *p* = 0.02). Compared to those without ARDS, patients with ARDS had higher severity scores assessed by the APACHE II (26 vs. 21, *p* < 0.001) and Non-pulmonary SOFA (9 vs. 7, *p* < 0.001).

### Outcomes in patients with ARDS

In-hospital mortality in patients with and without ARDS was 29.9% and 16.5%, respectively (*p* = 0.007) (Table 2). Of the 404 patients with septic shock, the in-hospital mortality rate of those with ARDS was significantly higher than that of patients without ARDS (32.8% vs. 17.9%, *p* = 0.01). The median ventilator-free days for patients with ARDS was less than that for patients without ARDS (15 [0–21] vs. 22 [9–28], *p* < 0.001), as was the median ICU-free days (14 [4–19] vs. 19 [10–24], *p* < 0.001). However, there was no significant difference in the length of hospital stay by ARDS status (25 [11–61] vs. 26 [15–51], *p* = 0.39). In terms of survivor dispositions, a larger proportion of patients with ARDS than without ARDS needed to be transferred to other facilities.

**Table 2:** Outcomes comparing patients with and without ARDS among patients with non-pulmonary sepsis

Variable		ARDS (*n* = 85)	No ARDS (*n* = 509)	*P* value
In-hospital mortality		23 (29.9)	84 (16.5)	0.007
	with septic shock (*n* = 404)	20 (32.8)	60 (17.9)	0.01
	Without septic shock (*n* = 190)	3 (18.8)	24 (13.9)	0.71
Survivor dispositions (*n* = 479)	Home	10 (18.5)	157 (36.9)	0.006
	Transfer	44 (81.5)	268 (63.1)	
ICU-free days		14 (3.75–19.25)	19 (10–24)	< 0.001
Ventilator-free days		15 (0–21)	22 (8.75–28)	< 0.001
Length of hospital stay		25 (11–61)	26 (15–51)	0.35

Reported counts (proportions) for categorical variables and median (interquartile range) for continuous variables

*ARDS* acute respiratory distress syndrome, *ICU* intensive care unit

Missing data (due to missing data of each outcome measures): in-hospital mortality = 8; survivor dispositions = 0; ICU-free days = 89; ventilator-free days = 9; length of hospital stay = 8

### Risk modifiers for having ARDS

In the multivariate logistic regression model, we identified three main risk modifiers associated with having ARDS (Table 3). Notably, the odds of having ARDS were higher for patients from the emergency department than for those transferred from hospital wards or other hospitals (OR, 1.89 [1.06–3.40]), for patients with soft tissue infection than for those with abdominal infection (OR, 2.37 [1.04–5.40]), and for those with a higher APACHE II score (OR, 1.08 [1.05–1.12]).

**Table 3:** Multivariable analysis for having ARDS associated with non-pulmonary sepsis (*n* = 594)

Variable		Odds ratio (95% CI)	*P* value
Age at admission—per year		0.99 (0.97–1.01)	0.29
Male gender		1.33 (0.71–2.49)	0.37
Admission source	Hospital wards and other hospitals	Reference	
	Emergency department	1.89 (1.06–3.40)	0.03
	Intensive care unit	0.96 (0.25–3.65)	0.95
Body mass index—kg/m^2^		1.04 (0.98–1.09)	0.19
Smoking	Never	Reference	
	Former	0.77 (0.39–1.54)	0.46
	Current	0.18 (0.06–0.59)	0.004
Coexisting conditions	Congestive heart failure	0.72 (0.26–1.88)	0.50
	COPD	1.54 (0.46–5.21)	0.49
	Diabetes mellitus	0.69 (0.35–1.37)	0.29
Regular medication	Glucocorticoids	0.48 (0.19–1.22)	0.12
	Statin	0.36 (0.10–1.24)	0.11
Site of infection	Abdomen	Reference	
	Urinary tract	0.71 (0.33–1.55)	0.39
	Soft tissue	2.37 (1.04–5.40)	0.04
	Other than abdomen, urinary tract, or soft tissue	1.57 (0.74–3.32)	0.24
Septic shock		1.43 (0.74–2.78)	0.29
APACHE II score—per point		1.08 (1.05–1.12)	< 0.001

*ARDS* acute respiratory distress syndrome, *CI* confidence interval, *COPD* chronic obstructive pulmonary disease, *APACHE II* acute physiology and chronic health evaluation II

Definition of categorical variables: other than abdomen, urinary tract, or soft tissue = central nervous system, intravenous catheter, osteoarticular, endocardium, wound, implant device, and others

Missing data (due to missing data of each outcome measures): admission source = 1; body mass index = 9; smoking = 45; APACHE II score = 53

## Discussion

In this retrospective cohort study of patients with non-pulmonary sepsis, admission route (from the emergency department rather than wards or other hospitals), disease severity (a higher APACHE II score), and infection site (soft tissue rather than abdominal infection) were risk modifiers for non-pulmonary septic ARDS. However, obesity, DM, statins, glucocorticoids, and shock were not statistically associated with ARDS.

Duration of onset from infection could be a valid risk modifier of ARDS in non-pulmonary sepsis. In our results, admission from the emergency department was related to having ARDS, and it is possible that both direct and indirect ARDS developed soon after or at the onset of sepsis [15, 31]. Thus, ARDS may not have occurred after time had passed from admission, and further studies are needed to investigate the timing of the onset of ARDS in non-pulmonary sepsis.

Site of infection also appeared to be a risk modifier for ARDS in non-pulmonary sepsis. One study showed that abdominal infection was related to with ARDS [23], and another study showed that soft tissue infection was related to without ARDS in population that included pulmonary infection [13, 32]. The correlation with indirect ARDS in most previous studies may have been attenuated because pulmonary infection is a major predisposing condition and few studies focused on non-pulmonary infection [2, 14, 15]. We showed that, when excluding this, soft tissue infection could be related to having ARDS. Not only pulmonary but also severe soft tissue infection could be a novel risk modifier. However, these patients were more likely to be admitted to wards instead of ICUs, presumably because shock was less common [33]. By limiting our cohort to ICUs, we may have introduced some bias. It is possible that our data for site of infection reflect only disease severity, despite controlling for severity using the APACHE II score and shock status. Pathogens beyond the site of infection may also be related to having ARDS, but our sensitivity analysis did not show a difference (Additional file 1: Table S1). Further studies are needed to confirm which infection site is more related to developing ARDS in patients with non-pulmonary sepsis.

We confirmed that the severity of non-pulmonary sepsis (APACHE II score) was related to having ARDS, consistent with the results in previous studies [2, 11, 13]. In this study, we did not exclude the possibility of the pulmonary parameter of the APACHE II score representing pre-existing ARDS in the emergency department. Thus, we performed a sensitivity analysis by changing the APACHE II score with the non-pulmonary SOFA score, and it showed similar results to the main analysis (Additional file 1: Table S2). However, having shock was not related to having ARDS in our population, indicating that having ARDS in non-pulmonary sepsis might be associated with the development of multiple organ failure instead of circulatory failure (shock) [2, 31]. Further studies are needed to determine organ failures that are more likely to occur with ARDS.

We did not show roles for obesity, DM, statins, and glucocorticoids which have been shown to be risk modifiers for ARDS in previous studies. Although they were risk modifiers in direct and indirect ARDS combined, the tendencies of the ORs were similar. Otherwise, obesity may not be a risk modifier of ARDS due to non-pulmonary sepsis because of mechanism is not the same [23, 25]. DM, statin use, and glucocorticoid use have been protective against ARDS in some clinical and basic research [20, 21, 34], but this has not been carried through to randomized clinical trials [35–37] and we found no benefits associated with the regular use of these medications. However, it is perhaps the lack of significance for the roles of obesity and smoking that was most unexpected.

Obesity is considered a risk modifier for ARDS because patients with obesity need higher tidal volumes, positive end-expiratory pressures, and sufficiently high peak airway pressures to counter the pressure of their chest wall and abdomen [16, 38]. The lack of difference in this study may reflect our small sample size.

Smoking has also been clearly linked as a direct risk modifier in clinical studies [17, 18], which is known to occur through direct damage to the alveolar epithelium that leads to local inflammation [39, 40]. Despite this, our results did not support it even indirectly, and we consider there to be two main reasons. First, smoking history may have been difficult to assess in critically ill patients. Including a combination of smoking-related biomarkers might have identified more current smokers than the smoking history obtained from patients, surrogates, and medical records [41]. Second, unrecorded medication histories, including the use of inhaled corticosteroids and inhaled beta agonist may have been a confounding factor [42]. It is conceivable that smoking and indirect ARDS are not associated, as is the case with smoking and direct ARDS [23, 25]. Since it is difficult to consider smoking as a protective factor, we only used smoking as an adjustment factor in this study.

Risk modifiers for ARDS among patients with non-pulmonary sepsis were similar to those reported for patients with direct and indirect ARDS in previous studies, but they were not the same. This information may help clinicians and researchers. For clinicians, it is important to carefully treat non-pulmonary sepsis particularly in patients with risk modifiers that we have shown. For researchers, it may help to develop future study design and may provide more research on which to assess risks. We recommend that more classifications or adjustments are needed for ARDS because of the large heterogeneity in the syndrome.

### Limitations

Several limitations of this study need to be acknowledged. First, we did not capture all ARDS episodes because we only performed screening on the first or fourth day. However, Most ARDS develops within 4 days of admission [2, 11, 15] and most cases occur within 12 h if sepsis is a predisposing condition [43]. Second, we only included patients in ICUs, although it should be noted that most cases would have been admitted to ICUs anyway [2]. Third, there could be some unmeasured confounders because of the post hoc analysis, despite using mostly the same factors as in previous studies [2, 11, 13]. Fourth, we diagnosed ARDS based on the application of the Berlin criteria by the physician in charge. Because the diagnosis of ARDS is difficult [44], some cases might not have been diagnosed correctly, even if they had respiratory failure. Fifth, we assessed risk modifiers at the first day of registration, yet we know that the value of some factors might be related to timing. However, risk modifier candidates were limited to patient backgrounds and characteristics, which were fixed at data collection. Sixth, based on the results of power analysis, the sample size of this study may not have been enough for the assessment of smoking and BMI as risk modifiers. Finally, our cohort was limited to Japan [2, 11, 13], and important geographic variations may have been missed [45].

## Conclusions

Our retrospective cohort study from the Japanese sepsis registry revealed that admission route, severity, and infection site could be risk modifiers for ARDS in patients with non-pulmonary sepsis.

## Supplementary information

**Additional file 1: Table S1.** Multivariable analysis including pathogens. **Table S2.** Multivariable analysis including Non-pulmonary SOFA score instead of APACHE II score. Two supplementary tables contain the results of sensitivity analyses indicated in the main manuscript.

## Data Availability

The datasets used and/or analyzed during the current study are available from the corresponding author on reasonable request.

## Abbreviations

APACHE: Acute Physiology and Chronic Health Evaluation
ARDS: Acute respiratory distress syndrome
FORECAST: Focused Outcomes Research in Emergency Care in Acute Respiratory Distress Syndrome, Sepsis and Trauma
ICUs: Intensive care units
OR: Odds ratio
