# Supplementary material for: Risk modifiers of acute respiratory distress syndrome in patients with non-pulmonary sepsis: a retrospective analysis of the FORECAST study
Source: J Intensive Care. 2020 Jan 10;8:7. doi: 10.1186/s40560-020-0426-9 (PMC6954566; doi:10.1186/s40560-020-0426-9)
Supplement: Supplementary file 1 — Additional file 1: Table S1. Multivariable analysis including pathogens. Table S2. Multivariable analysis including Non-pulmonary SOFA score instead of APACHE II score. Two supplementary tables contain the results of sensitivity analyses indicated in the main manuscript. [file 40560_2020_426_MOESM1_ESM.docx]

Additional file 1

Table S1: Multivariable analysis including pathogens

| Variable |  | Odds ratio  (95% CI) | P Value |
| --- | --- | --- | --- |
| Age at admission — per year |  | 0.99 (0.97–1.01) | 0.29 |
| Male gender |  | 1.30 (0.69–2.45) | 0.41 |
| Admission source | Hospital wards and transfer | Reference |  |
|  | Emergency department | 1.92 (1.06–3.48) | 0.03 |
|  | Intensive care unit | 0.91 (0.24–3.51) | 0.89 |
| Body mass index — per kg/m^2^ |  | 1.04 (0.98–1.10) | 0.18 |
| Smoking | Never | Reference |  |
|  | Former | 0.79 (0.39–1.58) | 0.50 |
|  | Current | 0.18 (0.06–0.58) | 0.004 |
| Coexisting conditions | Congestive heart failure | 0.72 (0.28–1.89) | 0.51 |
|  | COPD | 1.57 (0.47–5.30) | 0.47 |
|  | Diabetes mellitus | 0.67 (0.34–1.34) | 0.26 |
| Regular medication | Glucocorticoids | 0.48 (0.19–1.22) | 0.12 |
|  | Statin | 0.36 (0.10–1.25) | 0.11 |
| Suspected site of infection | Abdomen | Reference |  |
|  | Urinary tract | 0.67 (0.30–1.50) | 0.33 |
|  | Soft tissue | 2.56 (1.08–6.08) | 0.03 |
|  | Other than abdomen, urinary tract, or soft tissue | 1.68 (0.76–3.69) | 0.20 |
| Septic shock |  | 1.41 (0.73–2.74) | 0.31 |
| APACHE II score — per point |  | 1.08 (1.05–1.12) | < 0.001 |
| Pathogens | Gram positive coccus | 0.87 (0.43–1.77) | 0.70 |
|  | Gram negative rod | 1.16 (0.61–2.21) | 0.66 |
| Reported counts (proportions) for categorical variables and median (interquartile range) for continuous variables. | | | |
| Definition of abbreviations:  ARDS = acute respiratory distress syndrome; CI = confidence interval; COPD = chronic obstructive pulmonary disease; APACHE II = acute physiology and chronic health evaluation II. | | | |
| Definition of categorical variables:  Other than abdomen, urinary tract, or soft tissue = central nervous system, intravenous catheter, osteoarticular, endocardium, wound, implant device, and others; Pathogens = Positive blood culture without clinically confirmed contamination; Gram positive coccus = Staphylococcus, Streptococcus, and Enterococcus; Gram negative rod = Acinetobacter, Aeromonas, Burkholderia, Citrobacter, Escherichia, Enterobacter, Haemophilus, Klebsiella, Legionella, Pseudomonas, Proteus, Salmonella, Serratia, Stenotrophomonas, and Vibrio. | | | |
| Missing data (due to missing data of each outcome measures):  Admission source = 1; Body mass index = 9; Smoking = 45; APACHE II score = 53. | | | |

Table S2: Multivariable analysis including Non-pulmonary SOFA score instead of APACHE II score

| Variable |  | Odds ratio  (95% CI) | P Value |
| --- | --- | --- | --- |
| Age at admission — per year |  | 1.00 (0.98–1.02) | 0.75 |
| Male gender |  | 1.24 (0.69–2.21) | 0.47 |
| Admission source | Hospital wards and other hospitals | Reference |  |
|  | Emergency department | 2.23 (1.28–3.89) | 0.005 |
|  | Intensive care unit | 1.20 (0.39–3.71) | 0.75 |
| Body mass index — per kg/m^2^ |  | 1.04 (0.99–1.10) | 0.09 |
| Smoking | Never | Reference |  |
|  | Former | 0.86 (0.46–1.62) | 0.64 |
|  | Current | 0.20 (0.06–0.60) | 0.004 |
| Coexisting conditions | Congestive heart failure | 0.78 (0.34–1.79) | 0.56 |
|  | COPD | 1.51 (0.43–5.26) | 0.52 |
|  | Diabetes mellitus | 0.88 (0.48–1.62) | 0.68 |
| Regular medication | Glucocorticoids | 0.79 (0.35–1.78) | 0.58 |
|  | Statin | 0.35 (0.18–1.07) | 0.07 |
| Suspected site of infection | Abdomen | Reference |  |
|  | Urinary tract | 0.59 (0.28–1.24) | 0.17 |
|  | Soft tissue | 1.75 (0.81–3.77) | 0.16 |
|  | Other than abdomen, urinary tract, or soft tissue | 1.49 (0.75–2.94) | 0.26 |
| Septic shock |  | 1.27 (0.64–2.50) | 0.49 |
| Non-pulmonary SOFA score — per point |  | 1.16 (1.07–1.27) | < 0.001 |
| Reported counts (proportions) for categorical variables and median (interquartile range) for continuous variables. | | | |
| Definition of abbreviations:  ARDS = acute respiratory distress syndrome; CI = confidence interval; COPD = chronic obstructive pulmonary disease; SOFA = sequential organ failure assessment. | | | |
| Definition of categorical variables:  Other than abdomen, urinary tract, or soft tissue = central nervous system, intravenous catheter, osteoarticular, endocardium, wound, implant device, and others. | | | |
| Missing data (due to missing data of each outcome measures):  Admission source = 1; Body mass index = 9; Smoking = 45; Non-pulmonary SOFA score =46. | | | |
